# Assessing the feasibility of online SSVEP decoding in human walking using a consumer EEG headset

**DOI:** 10.1186/1743-0003-11-119

**Published:** 2014-08-09

**Authors:** Yuan-Pin Lin, Yijun Wang, Tzyy-Ping Jung

**Affiliations:** Swartz Center for Computational Neuroscience, Institute for Neural Computation, University of California, San Diego, La Jolla, CA USA; Center for Advanced Neurological Engineering, Institute of Engineering in Medicine, University of California, San Diego, La Jolla, CA USA

**Keywords:** EEG, Consumer-level EEG headset, SSVEP, BCI, Moving humans

## Abstract

**Background:**

Bridging the gap between laboratory brain-computer interface (BCI) demonstrations and real-life applications has gained increasing attention nowadays in translational neuroscience. An urgent need is to explore the feasibility of using a low-cost, ease-of-use electroencephalogram (EEG) headset for monitoring individuals’ EEG signals in their natural head/body positions and movements. This study aimed to assess the feasibility of using a consumer-level EEG headset to realize an online steady-state visual-evoked potential (SSVEP)-based BCI during human walking.

**Methods:**

This study adopted a 14-channel Emotiv EEG headset to implement a four-target online SSVEP decoding system, and included treadmill walking at the speeds of 0.45, 0.89, and 1.34 meters per second (m/s) to initiate the walking locomotion. Seventeen participants were instructed to perform the online BCI tasks while standing or walking on the treadmill. To maintain a constant viewing distance to the visual targets, participants held the hand-grip of the treadmill during the experiment. Along with online BCI performance, the concurrent SSVEP signals were recorded for offline assessment.

**Results:**

Despite walking-related attenuation of SSVEPs, the online BCI obtained an information transfer rate (ITR) over 12 bits/min during slow walking (below 0.89 m/s).

**Conclusions:**

SSVEP-based BCI systems are deployable to users in treadmill walking that mimics natural walking rather than in highly-controlled laboratory settings. This study considerably promotes the use of a consumer-level EEG headset towards the real-life BCI applications.

## Background

Bridging the gap between laboratory brain-computer interface (BCI) demonstrations and real-life applications has gained increasing attention nowadays in translational neuroscience. A recent brain imaging study in mobile humans suggested that the brain switches to a different operating method while humans actively behave, move, walk, and orient in ecologically-valid environments [1]. That is, there might be significant differences in how the brain works in ecologically valid environments versus highly controlled laboratory environments [2, 3]. However, studies linking brain dynamics to cognitive functions have been majorly devoted to the stationary and tethered individuals, in which the participants underwent the experiments with highly-controlled settings in laboratories. They were usually instructed to avoid any task-irrelevant head/body movements. The laboratory-oriented research might have a generalizability issue when it is deployed in the real world. Therefore, studying the brain dynamics associated with naturalistic human behaviors is of great importance in translational neuroscience.

Promising advances in mobile electroencephalogram (EEG) system have largely progressed in naturalistic brain imaging. Wearing a lightweight mobile EEG headset allows EEG recording under naturalistic movements. Several previous studies have proved the efficacy of using either experimental prototype [4–6] or consumer-level EEG headsets [7–13], *e.g.* the MindWave headset (NeuroSky, Inc.) and the Emotiv EEG headset (Emotiv systems, Inc.) in cognitive neuroscience, neuroimaging, and BCI domains. Nevertheless, until recently only scattered studies [14–18] employed such mobile brain sensing technology to explicitly assess EEG activities while participants freely and actively adapt to and interact with their naturalistic environments. Specifically, De Vos *et al.* [17] recently demonstrated the feasibility of a mobile auditory P300-based BCI system during natural walking. Nevertheless, the obtained BCI performance, *i.e.*, information transfer rate (ITR), needed to be improved to satisfy performance requirement in practical applications.

The steady-state visual-evoked potential (SSVEP)-based BCI has become a popular communication channel that allows users to interact with environments/external devices due to its ease of use, minimal user training, large number of commands and high ITR [19–21]. However, most of previous works were rooted in the SSVEP correlates of stationary and tethered subjects who were instructed to attentively gaze at the visual stimulation and avoid other gross movements. Recently, sparse works which adopted a laboratory-grade EEG device focused on an offline assessment of the SSVEP dynamics during walking [15, 18]. Alternatively to the use of lab-graded headgear, few studies noticed the need of realizing an SSVEP-based BCI using the consumer-level headset [11–13]. Yet, these studies tested the efficacy with highly movement-controlled subjects. No study has reported the test of the consumer EEG headset for actuating the online SSVEP-based BCI with natural head/body positions and movements, *i.e.*, in non-stationary, non-tethered subjects.

The main objective of this study was to assess whether or not a consumer-level headset can be used to perform online SSVEP decoding in human walking. To this end, the subjects were instructed to stand or walk on a speed-controllable treadmill (0.45, 0.89, and 1.34 meters per second (m/s)) in an attempt to initiate different levels of walking locomotion while using the online BCI. As compared to the standing condition, the methods and empirical results of moving subjects could shed the light on principles regarding the utility of a user-friendly and affordable commercial EEG headset for implementing real-life BCI applications towards naturalistic environments.

## Methods

### Participant

Seventeen healthy participants (14 males and 3 females; 22–32 years of age; mean age, 26.76 years) with normal or corrected-to-normal vision participated in this study. They were randomly selected from the student volunteers at University of California San Diego (UCSD). UCSD Human Research Protections Program approved this study. Each participant read and signed an informed consent before the experiment.

### EEG recording

This study employed a 14-channel Emotiv EEG headset that sampled EEG signals at 128 Hz and band-pass filtered them between 0.2 and 45 Hz. The electrodes were positioned at AF3, F7, F3, FC5, T7, P7, O1, O2, P8, T8, FC6, F4, F8, and AF4 in accordance with the modified international 10–20 system. In addition, the angular velocities were concurrently recorded by the built-in two-channel gyroscope (Gyro-X: horizontal movement; Gyro-Y: vertical movement) in order to characterize the severity of the head movements during walking. The Emotiv Control Panel software provides visual monitoring of the electrode impedance. Once all electrodes had good contacts with the scalp, a laptop (Thinkpad X230, Lenovo Inc.) started to initiate the OpenViBE software [22] for connecting to the Emotiv headset, and then activate the BCILAB toolbox [23] to stream EEG signals for real-time frequency detection of the SSVEP signals.

### Treadmill experiment setup

**Figure 1:**
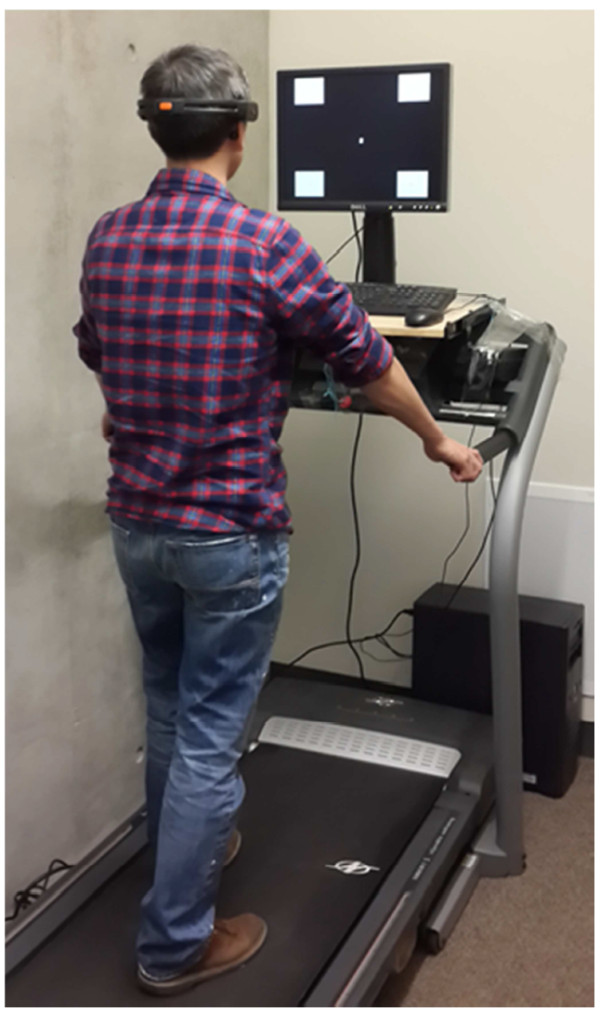
**The illustration of experiment setup for SSVEP recordings.**

This study implemented a gaze-dependent SSVEP decoding system, which needed users to shift their visual focus to visual targets to elicit SSVEPs [21]. This study instructed participants to stand or walk on a treadmill at speeds of 0.45, 0.89, and 1.34 m/s (*i.e.*, 1, 2, and 3 mile(s) per hour (MPH)) and intentionally gazed at a fixation cross or one of four repetitive black/white visual flickers (frequency: 9 Hz, 10 Hz, 11 Hz, and 12 Hz; size: 7 cm × 6 cm) concurrently presented on a 19-inch LCD monitor with a 60 Hz refresh rate (Figure 1). The paradigm can be used to implement a four-direction cursor control system. The generation of visual stimuli conformed to the approach that can be used to elicit SSVEPs at a flexible frequency [6]. The horizontal and vertical intervals between two stimuli were 19 cm and 17 cm respectively. The monitor was placed on a stand mounted above the control panel of the treadmill and adjusted to the same height as the participants’ eyes. The participants were instructed to hold the treadmill hand grip during standing and walking to maintain a constant viewing distance (~60 cm) between the subjects’ eyes and the monitor. Each subject participated in four sessions (each corresponded to one of four walking speeds from standing, 0.45 m/s, 0.89 m/s to 1.34 m/s) on the same day. Each session repeated a run 10 times. In each run, a verbal cue guided participants to shift their attention on the five targets (*i.e.*, fixation cross and four flickers) sequentially. The participants were instructed to fixate at the fixation cross for three seconds and then shift their gaze to the flicker within 1.5 seconds after the verbal cue. The averaged time for detecting each target was 4.25 seconds (see below for more details on SSVEP detection). Each run lasted ~26 seconds in total. Thus, each session lasted around 4.3 minutes. Note the data within the periods of target-shifting were ignored in online SSVEP detection, and data recorded as participants gazed at the fixation cross were only used as baseline control in offline analysis. Each participant underwent a 4-session experiment (in the order of standing 0.45 m/s, 0.89 m/s to 1.34 m/s without randomization) with a minute(s) between-session rest to prevent visual and/or motor fatigue. During the rest, the signal qualities of electrodes were re-checked and adjusted via the Emotiv Control Panel. That is, without the need of removing the headset a syringe was used to drip a little more saline solution to the sponges of the electrodes with poor conductivity. The entire experiment for four sessions and between-session rests lasted less than 30 minutes.

### Online SSVEP detection

The 14-channel EEG data in the 0.2-45 Hz frequency range (Emotiv default setting) were submitted to the process of frequency detection based on canonical correlation analysis (CCA) [19, 24] which has been proved highly efficient for enhancing the signal-to-noise ratio (SNR) of SSVEP signals [18, 19, 24]. CCA is a multivariate statistics method to maximize underlying correlation between two given multivariate signals *x* and *y.* To this end, CCA seeks the weight vectors *w*_*x*_ and *w*_*y*_ to project the input signals into *X* and *Y,* respectively, leading to the maximum canonical correlation *ρ* according to the following equation:


In this experiment, *x* refers to the *n-*second EEG signals (*n* is a self-regulated variable described below), and *y* refers to a set of reference signals generated as follows:


where *f* and *f*_s_ are the target and sampling frequency, respectively. *N*_*h*_ is the number of harmonics, and *N* is the number of sampling points. The CCA calculates the canonical correlation between the given EEG signals *x* and the reference signals *y* at each target frequency. The final decision of SSVEP frequency was made upon the maximum correlation among the reference signals. This study set the target frequency *f* as 9, 10, 11 and 12 Hz and the *N*_*h*_ as two for generating template signals based on fundamental frequency and second harmonics with respect to the target frequency. Particularly, this study adopted the procedure of self-regulating data length [21] to sequentially accumulate the acquired EEG signals for improving the SNR of SSVEPs. This process used EEG data within several windows ranging from 2 to 8 seconds, with an increment of 0.25, for CCA calculation (*e.g.*, 2, 2.25, 2.5… 8 s windows). Only if the detected frequency of the SSVEPs within four consecutive windows matched the target frequency, the detection pipeline would consider the target correctly classified. This detection pipeline was similar to the idea used in an adaptive P300 BCI [25]. Otherwise, the target would terminate as an incorrect decision. Note that, to eliminate the distraction from online feedback, this study did not present feedback to the subjects. The detection accuracy, decision time and ITR [26] were used to evaluate the SSVEP decoding performance. This study also saved the EEG data during the online experiments for further offline analysis.

### Offline data analyses

The offline analyses aimed to explore the dynamics of the background EEG activity and the SSVEP amplitude acquired by the consumer headset under different walking speeds. The background EEG spectrum in the fixation-cross condition, which can be regarded as baseline power for the SSVEP conditions under each walking speed, was necessary to reveal the underlying brain activity associated with the walking locomotion. In addition, since this study adopted treadmill walking to systematically test the effects of different degrees of head movements on the SSVEPs, it is important to report the intensity and frequency of the head movements. To this end, we applied the spectrum analysis to the 2-channel gyroscope signals (horizontal and vertical axes) along different walking speeds. The detailed steps are described as follows. This study applied a 1 Hz high-pass finite impulse response (FIR) filter to the EEG signals to remove low-frequency drifts, resulting in a signal bandwidth of 1 to 45 Hz for the spectral analysis. The 128-point short-time Fourier transform (STFT) with a Hamming window of length 128 samples and 25% overlap was then applied to estimate the EEG spectrogram at a 1 Hz frequency resolution. The grand average power spectral density (PSD) of each channel can be derived for each condition. Note that, to dissociate the reactive SSVEP from the background EEG activities and walking related noises, this study estimated the relative power of SSVEPs by subtracting the power spectrum in the fixation-cross condition from those of the SSVEP conditions at each of the walking speeds. In addition, this study calculated the scalp distributions of the spectral power to form topographic maps using the EEGLAB toolbox [27]. The spectrogram of the recorded 2-channel Gyro signals was estimated using the same procedure as for EEG data (but without filtering). Lastly, this study employed a paired *t*-test to access the differences of SSVEP amplitudes and online performance between two walking conditions (standing, 0.45 m/s, 0.89 m/s, and 1.34 m/s).

## Results

### The impacts of walking movements on the EEG spectra

**Figure 2:**
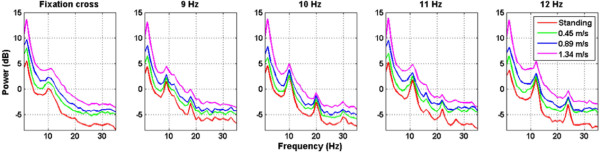
**The average power spectral density of O1 and O2 channels across 17 subjects in response to the fixation-cross and visual targets flickering at 9, 10, 11, and 12 Hz while standing still or walking on the treadmill with the speeds of 0.45, 0.89, and 1.34 m/s.**

**Figure 3:**
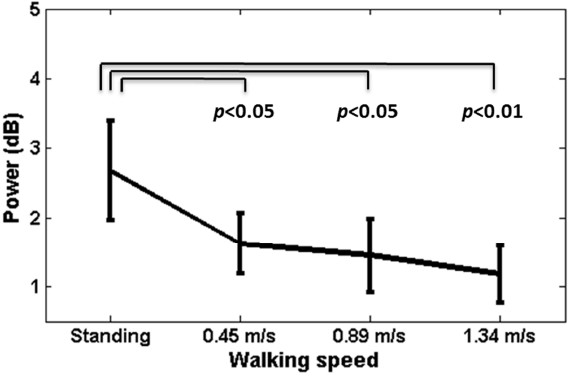
**The averaged power of SSVEP signals at O1 and O2 channels across 17 subjects under different walking speeds after alleviating the wideband noise by subtracting the power in the fixation-cross condition from that of the SSVEPs in the flickering conditions.** The thin lines indicate the significant difference between speeds (*p* < 0.05 or < 0.01), whereas the error bars represent the standard error of the SSVEP amplitudes.

Figure 2 shows the averaged PSD of O1 and O2 channels across all subjects. In general, walking locomotion induced wideband noise to the EEG spectra, very likely arising from electrode shift with respect to the scalp and electromyography (EMG) activities from facial, neck, and scalp muscles during walking. This phenomenon led to power lifting proportional to walking speeds. Fast walking especially induced large augmentation of low-frequency power around 2 Hz. In addition, the SSVEP amplitudes under the standing and walking conditions exhibited salient peaks at the fundamental and second harmonics of the flickering frequencies, which appeared to monotonically attenuate as walking speed increased. To estimate SSVEP amplitudes under different walking speeds, this study alleviated the wideband noise by subtracting the power in the fixation-cross condition from that of the SSVEPs in the flickering conditions. Figure 3 shows that the SSVEP signals at the O1-O2 pair declined evidently as walking speed increased, leading to significant amplitude drops (*p* < 0.05) between standing versus walking (0.45, 0.89, and 1.34 m/s). No significant differences were found between the three walking speeds.

**Figure 4:**
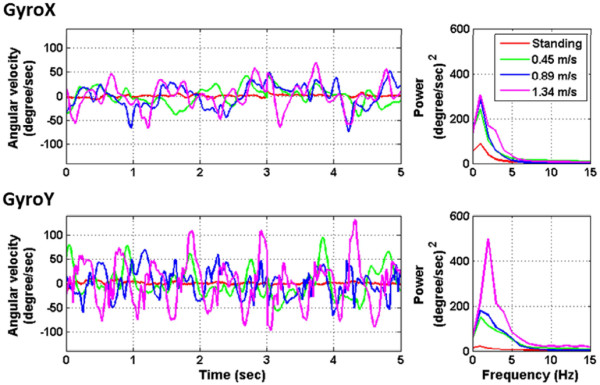
**The left panel illustrates an example of 5-second time course of 2-channel gyroscope signals (GyroX: horizontal plane, GyroY: vertical plane) associated with different walking speeds from one representative subject.** The right panel shows the averaged spectral profiles of 17 subjects across all stimulus conditions under different speeds.

Figure 4 illustrates an example of 2-channel gyroscope time course of a representative subject and averaged spectral profiles of 17 subjects across all conditions along the horizontal (Gyro-X) and the vertical (Gyro-Y) planes associated with walking locomotion under different walking speeds. As can be seen, compared with the standing condition, the low-frequency power (1 to 5 Hz) of the angular-velocity deviations tended to progressively augment as walking speed increased. Especially, the vertical head displacements (Gyro-Y) showed a big jump at 2 Hz while walking at 1.34 m/s. This result could be attributed to the fact that natural walking posture was typically accompanied by head bobbing (Gyro-Y) and head swaying (Gyro-X) while fast walking exhibited bigger head bobbing than head swaying.

**Figure 5:**
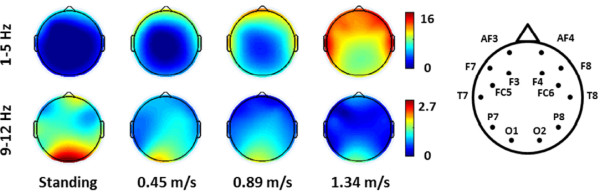
**The topographical mapping of the relative low-frequency power (1–5 Hz) and SSVEP amplitude (9–12 Hz) (derived from flickering minus fixation-cross) under different walking speeds.** The topographic legend indicates the channel locations of the 14-channel Emotiv EEG headset. The units of color bars represent the original EEG power in the dB scale. Note that these 14-channel interpolated maps (with the absence of the centro-parietal electrodes) do not imply spectral changes in the centro-parietal regions during walking.

Figure 5 plots the topographic maps of the relative low-frequency power (1–5 Hz) and the SSVEP amplitudes (9–12 Hz) (derived from flickering minus fixation-cross) under different walking speeds. As aforementioned, the head movements during walking resulted in power increase at frequencies from 1 to 5 Hz. The topographic maps showed that the EEG power augmentations, which mainly distributed over the fronto-central areas versus the posterior areas, more and less positively correlated with the waking speed. Furthermore, the SSVEP maps clearly showed that the induced occipital SSVEP amplitudes tended to be weaker during fast walking. It is worth noting that since the topographic spectrum maps were interpolated on a sparse 14-channel montage with the absence of centro-parietal electrodes, the results did not lead to the implication of spectral changes in the sensorimotor cortex during walking.

### The online SSVEP detection performance

**Figure 6:**
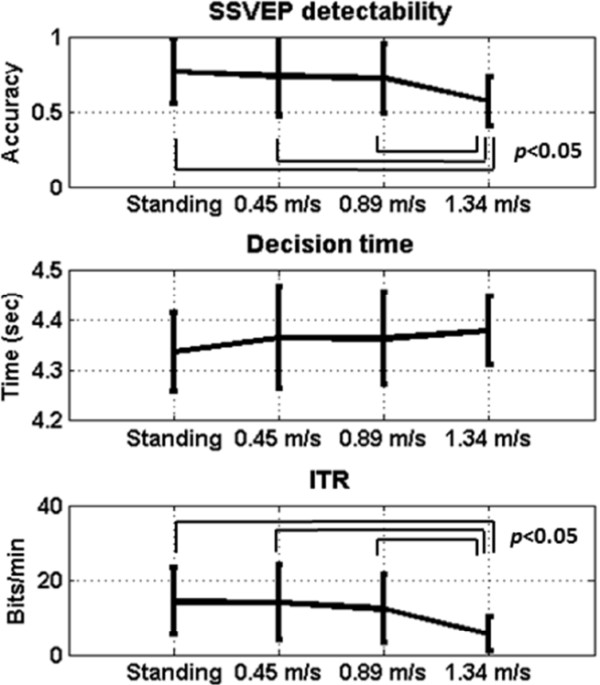
**The online SSVEP decoding results in terms of averaged accuracy (%), decision time (sec), and ITR (bits/min) at the different walking speeds.** The error bars represent the standard deviation of the online results.

Figure 6 shows the online SSVEP decoding performance in terms of detection accuracy (%), decision time (sec), and ITR (bits/min) at different walking speeds. In general, the classification accuracy was significantly higher than the chance level (25%) under all conditions. The averaged BCI performance progressively decayed as walking speed increased from 0.45 to 1.34 m/s (accuracy: 73.49 ± 26.52%, 71.83 ± 23.17%, 56.57 ± 16.56%; ITR: 14.04 ± 10.10, 12.37 ± 9.19, 5.53 ± 4.57; with the representation of the mean value plus/minus standard deviation), as compared to the standing condition (accuracy: 76.60 ± 21.74%, ITR: 14.38 ± 9.04). The significant drops (*p* < 0.05) in accuracy and ITR were only found between the 1.34 m/s condition versus the other three conditions. The decision time was found to slightly increase as walking speed increased, but there was no significant difference (*p* > 0.05) between speeds (standing: 4.34 ± 0.08 s; 0.45 m/s: 4.37 ± 0.10s; 0.89 m/s: 4.36 ± 0.09 s; 1.34 m/s: 4.38 ± 0.07 s).

## Discussion

This study aimed to assess whether or not a consumer-level headset can be used to perform online SSVEP decoding for users in naturalistic positions, postures and movements. To this end, this study preliminarily tested the validity of an online four-target SSVEP decoding system outside standard laboratories by using a speed-controlled treadmill experiment that generated different degrees of head movements in moving humans. The online performance appeared to decrease as participants switched from standing to walking. A significant drop (*p* < 0.05) in accuracy and ITR was found during fast walking at 1.34 m/s (*c.f.* Figure 6). The reason might be attributed to the fact that the SSVEP amplitude was negatively correlated with the walking speed (*c.f.* Figures 3 and 5). The amplitude of SSVEP has been found to be largely modulated by visual spatial attention [28]. As a consequence, the loss of focus could reduce visual attention and thereby lead to the decreased SSVEP amplitude. This very likely happened in treadmill walking. Fast walking obviously initiated rapid head bouncing especially in fast walking (*c.f.* Figure 4). Such phenomenon was also confirmed by the participants of this study reported somehow more difficult in focusing on the flickering targets during walking, especially at the speed of 1.34 m/s. However, since the treadmill walking protocol adopted in this study did not randomize the walking speeds, we cannot completely rule out the possibility that the visual- or motor-fatigue could also contribute to the SSVEP deterioration. Currently, little is known about the interference of fatigue on SSVEP amplitude in an SSVEP experiment where multiple sessions are separated by inter-session breaks. A further study is needed to address the impact of fatigue on the BCI performance, which might likely happen in real-life applications.

Another interesting finding was that the increase of the gait cadence in our study (up to 1.34 m/s) accompanied larger head bobbing as well as moderate head swaying, resulting in head movements of 1–5 Hz (*c.f.* Figure 4). Such head movements inevitably swayed the headset and yielded a corresponding low-frequency power fluctuation spreading over the whole head (*c.f.* Figure 5), which was analogous to the evidence that gait-related artifacts significantly affected the EEG spectral power in the 1.5-8.5 Hz frequency range during walking (below 1.25 m/s) and running (1.9 m/s) [3]. Such 1–5 Hz mechanical artifacts produced by head movements during steady walking (tested up to 1.34 m/s in this study) should not affect the SSVEPs (9–12 Hz frequency range). In this regard, methods for movement artifact suppression [3] might not be able to improve the SSVEP detection performance in this study. However, the artifacts could contaminate other event-related potentials (ERPs) used in other types of BCIs. For example, P300 waveform is majorly composed of frontal theta (4-7 Hz) and posterior delta activations (<4 Hz) [29]. This gait-related low-frequency contamination could cause a deterioration of P300 quality during naturally walking [14, 17].

Lastly, one might concern the adopted treadmill-walking protocol prone to constrain the walking locomotion. This study instructed the subjects to hold the hand-grip while walking on the treadmill (*c.f.* Figure 1). It was not only to insure the subjects’ safety while proceeding with fast walking on the moving belt (1.34 m/s), but also to maintain their viewing distances to the display monitor while walking. Such hand-grip holding might result in dissimilar walking locomotion with less body and head movements compared to natural walking patterns in daily life. However, according to the measurements from the gyroscope (*c.f.* Figure 4), the treadmill-initiated walking locomotion evidently engaged pronounced head movements as compared to a standing posture. Unlike the previously laboratory-oriented BCI demonstrations with stationary subjects, this study was a valuable step towards the transition to real life applications.

Although the empirical results in this study demonstrated the applicability of using the consumer-level Emotiv headset to decode SSVEPs in moving humans, the obtained satisfactory performance might not assure that the headset could be perfectly suitable for all BCI applications. First, given the sparse 14-channel montage, the unavailability of midline and centro-parietal regions might hinder the measurements of good-quality P300 waveforms [14]. Second, the EEG quality measured by the designed saline electrodes highly depends on the moisture level of saline liquid, leading to progressive quality deterioration in a long-term use. Third, unlike the non-prep, dry electrodes [4], the pads of the saline electrodes might meet difficulties in having good contacts to hair-covered sites on the scalp. However, despite these potential drawbacks and inferior signal quality compared to medical-/laboratory-grade devices [30], the advantage of ease-to-use still could facilitate the BCI applications for non-critical applications in entertainment and game control.

Future efforts can be devoted to improve the online performance for freely moving humans. Wei *et al.* [31] recently proposed a new SSVEP detection method, namely differential canonical correlation analysis (dCCA), to improve the detectability of high-frequency SSVEPs as compared to the standard CCA. Thus, a natural next step is to employ the dCCA to improve the effectiveness of the SSVEP decoding. Another direction is to incorporate the consumer-level EEG headset with a head-mounted display device (presenting visual stimuli) to establish ubiquitous mobile BCI systems in ecologically valid environments.

## Conclusions

This study implemented the first online SSVEP decoding system in walking humans using a low-cost, easy-to-use commercial EEG headset, providing principles towards translating laboratory-oriented BCI demonstrations to practical BCI applications in daily life. The empirical results showed that the Emotiv EEG headset enabled SSVEP decoding with ITRs above 12 bits/min during slow walking (below 0.89 m/s). The ITR reported in the present study is comparable to the previous studies with stationary subjects. For example, a four-class SSVEP-based BCI speller using a commercial EEG system obtained an average ITR of 13 bits/min across a total of 106 subjects [32]. This study evidently demonstrated that SSVEP-based BCI systems are deployable to users in natural head/body positions and movements rather than in severely restrict movements within highly controlled laboratory environments.

## Abbreviations

BCI: Brain-computer interface
CCA: Canonical correlation analysis
dCCA: Differential canonical correlation analysis
EEG: Electroencephalography
ERP: Event-related potential
FIR: Finite impulse response
ITR: Information transfer rate
MPH: Miles per hour
PSD: Power spectral density
SNR: Signal-to-noise ratio
SSVEP: Steady-state visual-evoked potential
STFT: Short-time Fourier transform
m/s: Meters per second
UCSD: University of California San Diego.
